# Vehicle Detection by Fusing Part Model Learning and Semantic Scene Information for Complex Urban Surveillance

**DOI:** 10.3390/s18103505

**Published:** 2018-10-17

**Authors:** Yingfeng Cai, Ze Liu, Hai Wang, Xiaobo Chen, Long Chen

**Affiliations:** 1 Automotive Engineering Research Institution, Jiangsu University, Zhenjiang 212013, China; caicaixiao0304@126.com (Y.C.); xbchen82@gmail.com (X.C.), chenlong@ujs.edu.cn (L.C.); 2 School of Automotive and Traffic Engineering, Jiangsu University, Zhenjiang 212013, China; liuzee1314@foxmail.com

**Keywords:** Vehicle detection, traffic surveillance, deformable part model (DPM), conditional random field (CRF), context-based inference

## Abstract

Visual-based vehicle detection has been studied extensively, however there are great challenges in certain settings. To solve this problem, this paper proposes a probabilistic framework combining a scene model with a pattern recognition method for vehicle detection by a stationary camera. A semisupervised viewpoint inference method is proposed in which five viewpoints are defined. For a specific monitoring scene, the vehicle motion pattern corresponding to road structures is obtained by using trajectory clustering through an offline procedure. Then, the possible vehicle location and the probability distribution around the viewpoint in a fixed location are calculated. For each viewpoint, the vehicle model described by a deformable part model (DPM) and a conditional random field (CRF) is learned. Scores of root and parts and their spatial configuration generated by the DPM are used to learn the CRF model. The occlusion states of vehicles are defined based on the visibility of their parts and considered as latent variables in the CRF. In the online procedure, the output of the CRF, which is considered as an adjusted vehicle detection result compared with the DPM, is combined with the probability of the apparent viewpoint in a location to give the final vehicle detection result. Quantitative experiments under a variety of traffic conditions have been contrasted to test our method. The experimental results illustrate that our method performs well and is able to deal with various vehicle viewpoints and shapes effectively. In particular, our approach performs well in complex traffic conditions with vehicle occlusion.

## 1. Introduction

Collecting and generating vehicle information in an urban traffic monitoring system is a fundamental task of intelligent transportation systems (ITSs). Nowadays, as a result of the rapid pace of urbanization, vision-based vehicle detection faces great challenges. In addition to the challenges of outdoor visual processing, such as illumination changes, poor weather conditions, shadows, and cluttered backgrounds, vehicle detection by a stationary camera is further hindered by its own unique challenges, including various appearances and vehicle poses, and partial occlusion due to the loss of depth information or to traffic congestion.

**Figure 1:**
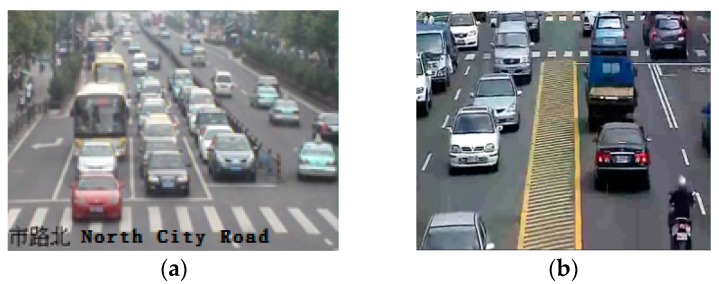
Two urban traffic conditions. (**a**) Urban traffic conditions 1; (**b**) Urban traffic conditions 2.

In fact, heavy traffic congestion has become common in many large cities, and as a result, many vehicle detection approaches that apply motion information to detect vehicles are not suitable, because the congestion causes vehicles to slow down, which reduces their motion (see Figure 1 for an example). During the past decade, vision-based object detection has been formulated as a binary classification problem with the goal of separating a target object from the background, and many of these algorithms achieve good performance with robust object detection.

Among the various binary classification approaches, the deformable part model (DPM) has been regarded with increasing interest. Evaluated using PASCAL Visual Object Classes (VOC) challenge datasets [1], DPM achieved state-of-the-art results in average precision (AP) for vehicle detection on the 2010 and 2011 benchmarks [2]. Since objects are detected as deformable configurations of parts, DPM should perform better at finding partially occluded objects; however, that performance has not been fully demonstrated for vehicle detection. Moreover, fusing semantic scene information by DPM methods, particularly in congested and complex traffic conditions, needs to be improved.

Motivated by prior work on deformable part models, mixtures of DPMs [3], and scene modeling [4], this paper proposes a probabilistic framework for vehicle detection based on fusing the results of structured part models and viewpoint inference. As the configuration of vehicle parts varies greatly as a function of monitoring viewpoints, structured part models are learned for each possible viewpoint using the part detection results. On the other hand, potential viewpoints of vehicles could be predicted by spatial context–based inference as vehicle motion is constrained by road structures and traffic signals.

**Figure 2:**
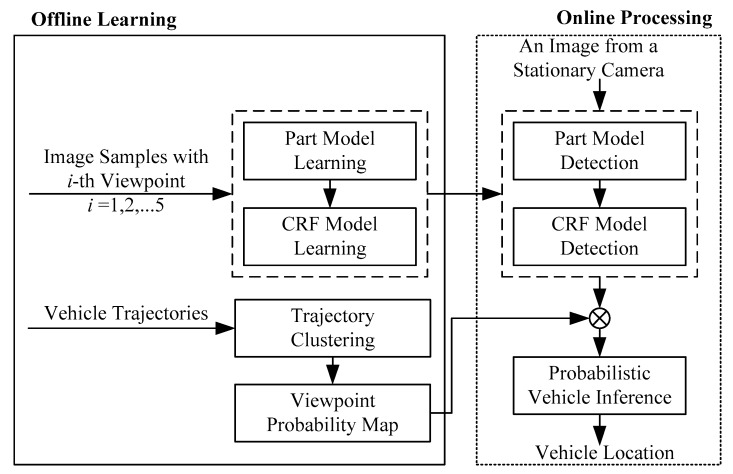
Framework of the proposed vehicle detection method. CRF, conditional random field.

The framework is depicted in Figure 2, which consists of two parts, offline learning and online processing. In this research, all the possible viewpoints and occlusion states for the stationary video cameras are summarized and the viewpoint-related discriminative part models are learned using an existing approach, described in [5]. All possible viewpoints in a certain location are generated by spatial context–based inference, where the logic constraint is obtained by trajectory analysis. This research extends DPM-based vehicle detection to multiview DPMs within a single probabilistic framework, which greatly improves the overall efficiency of vehicle detection. Such a method could be extended to on-road vehicle detection.

The remainder of this paper is organized as follows. In Section 2, an overview of the related work is given. The online probabilistic framework of vehicle detection is proposed in Section 3 and the offline discriminative part model learning is described in Section 4. Viewpoint detection based on spatial context inference is proposed in Section 5. Experimental results and analysis are presented in Section 6, followed by a conclusion and recommendations for future work in Section 7.

## 2. Related Works

Vision-based vehicle detection is widely used in ITSs in many parts of the world. Moving object detection methods are applied in many simple background environments, such as bridges, highways, and urban expressways. These methods can be described or defined as background modeling, frame differencing, and optical flow. They all have the ability to handle slight illumination changes; however, all of the techniques exhibit some performance limitations, as they are unable to detect stationary vehicles and incorrectly classify some moving objects as moving vehicles. Moreover, in relation to the congestion issue for urban surveillance, these methods may incorrectly identify several closely spaced vehicles as a single vehicle, or may not be able to detect any vehicle due to the lack of motion in a congested scene. Therefore, some research efforts attempt to utilize visual features of vehicles to detect them. 

Simple features such as color, texture, edge, and object corners are usually used to represent vehicles. Then the features are provided to a deterministic classifier to identify the vehicles [6,7,8,9]. Due to the unavailability or unreliability of some classification features in some instances, such as partial object occlusion, the utility of these methods can be limited to specific applications. 

Many recent studies on part-based models have been conducted to recognize objects while maintaining efficient performance under occluded conditions [10,11]. Using these methods, a vehicle is considered to be composed of a window, a roof, two rear-view mirrors, wheels, and other parts [12,13]. After part detection, the spatial relationship, motion cues, and multiple part models are usually used to detect vehicles [14]. Wang et al. [15] applied local features around the roof and two taillights (or headlights) to detect vehicles and identify partial occlusion. Li et al. proposed an AND-OR graph method [16,17] to detect front-view and rear-view vehicles. Saliency-based object detection has also been proposed [18]. 

Instead of manually identifying vehicle parts, they can be identified and learned automatically using a deformable part-based model. DPM was first proposed by Felzenszwalb [1] and achieved state-of-the-art results in PASCAL object detection challenges before the deep learning framework appeared, which had low real-time performance. Variants of this pathfinding work have been proposed by many subsequent research efforts. Niknejad et al. [19] employed this model for vehicle detection in which a vehicle is decomposed into five components: front, back, side, front truncated, and back truncated. Each component contained a root filter and six part filters, which were learned using a latent support vector machine and a histogram of oriented gradients features. In [5], the DPM was combined with a conditional random field (CRF) to generate a two-layer classifier for vehicle detection. This method can handle vehicle occlusion in the horizontal direction; however, experiments using artificial occlusion samples might suggest limited effectiveness of their approach in real-world traffic congestion conditions. In particular, in order to handle partial occlusions, various approaches were proposed to estimate the degree of visibility of parts, in order to properly weight the inaccurate scores of root and part detectors [20,21,22,23]. Wang et al. [24] proposed an on-road vehicle detection method based on a probabilistic inference framework. The relative location relationships among vehicle parts is used to overcome the challenges from multiview and partial observation. 

Additionally, some researchers directly built occlusion patterns from annotated training data to detect occluded vehicles. Pepikj et al. [25] modeled occlusion patterns in specific street scenes with cars parked on either side of the road. The established occlusion patterns demonstrated the ability to aid object detection under partially occluded conditions. Wang et al. [26] established eight types of vehicle occlusion visual models. The suspected occluded vehicle region is loaded into a locally connected deep model of the corresponding type to make the final determination. However, it is hard to establish a comprehensive set of all possible occlusion patterns in real-world traffic scenes.

Although the above approach effectively handles localized vehicle occlusion, more sophisticated methods are still needed for complex urban traffic conditions that are severely occluded between vehicles or multiple nonvehicle objects. While deformable part models have become quite popular, their value has not been demonstrated in video surveillance by stationary cameras. Meanwhile, by observation, it is found that for a specific monitoring scene, the vehicle motion pattern corresponding to road structures can be obtained with offline learning, and the obtained vehicle motion pattern can be used in deformable part models to enrich and improve the vehicle detection performance. In this paper, we summarize all the possible viewpoints and occlusion states in stationary video cameras, train the structured part models for each viewpoint by combining DPM with CRF in [5] to handle occlusion, and propose a probabilistic framework addressing the spatial contextual inference of viewpoint and part model detection results. The pros and cons of above related work are concluded in Table 1.

## 3. Online Probabilistic Framework 

**Figure 3:**
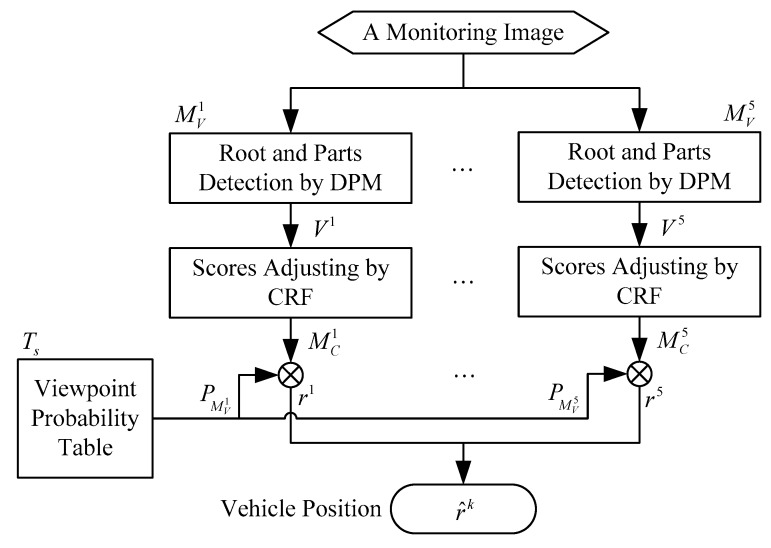
Online procedure of vehicle detection. DPM, deformable part model.

Given a frame in a traffic video, the online vehicle detection procedure is depicted in Figure 3. For each dominant viewpoint $M_{V}^{i}$, the features vector $v^{i}$ is generated by the DPM, which includes the scores of root, part filters, and best possible placement of parts. The viewpoint-related CRF is treated as the second-layer classifier, which uses the information from the DPM to detect the occluded vehicles. The detection conditional probability $M_{C}^{i}$ and location are output by the CRF. As the viewpoint is a location-specific parameter, this work uses a table-checking method to obtain the probability of a certain viewpoint $P_{M_{V}^{i}}$. In this research, i=1,2,…,5 are described in detail in Section V. The problem of vehicle detection can be represented by the following framework:

(1) $${\hat{r}}^{k}=\underset{r^{k}}{\arg\max}\;p(r^{k}|M_{C}^{k},T_{s})\;$$

where $r^{k}$ is the estimated location of a vehicle center for viewpoint k, $T_{s}$ is the knowledge of scene information, and $P_{M_{V}^{k}}=p(r^{k}|T_{s})$. The objective is to find the $r^{k}$ that maximizes $p(r^{k}|M_{C}^{k},T_{s})$. For the viewpoint from a fixed location that can be treated as independent from vehicle detection, the estimation can be converted to

(2) $$p(r^{k}|M_{C}^{k},T_{s})=Z[p(r^{k}|M_{C}^{k}),p(r^{k}|T_{s})]\;$$

where Z[a,b] is an adjustment function to avoid the effect of error of viewpoint probability. If b<a, Z[a,b]=a, otherwise Z[a,b]=a⋅b.

Below, the training of structured part-based vehicle models including DPM and CRF is given, and the estimation to $p(r^{k}|M_{C}^{k})$ of Equation (2) is described in Section 4. Typical types of road structures and the method of generating the possible viewpoints table will be defined, and the estimation to $p(r^{k}|T_{s})$ of Equation (2) will be given in Section 5.

## 4. Structured Part-Based Vehicle Models

**Figure 4:**
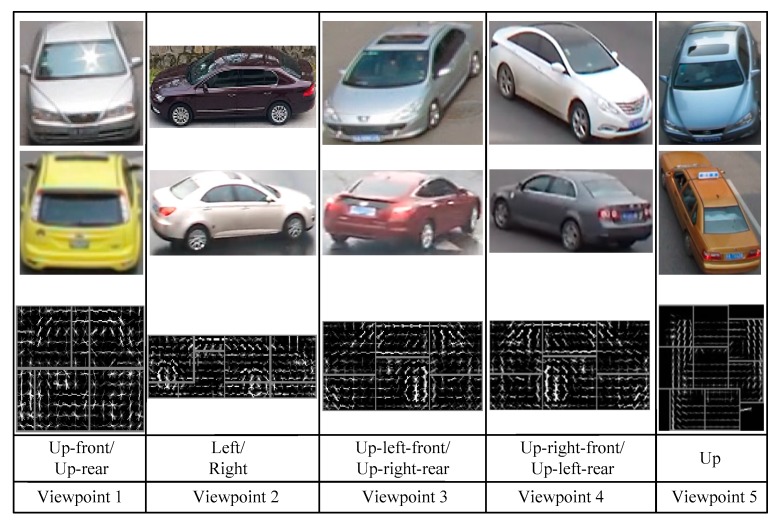
Five possible viewpoints of a vehicle as observed by a stationary camera.

The viewpoints of vehicles are closely related to the spatial contextual information of a traffic scene and the effect of possible occlusion states. In this paper, a summary of all possible vehicle viewpoints from the fixed camera location is proposed (as shown in Figure 4). Since cameras are usually installed at a certain distance above the ground, vehicle images for front viewpoints and rear viewpoints are similar in their visual feature space [17]. Similarly, this same observation yields four additional pairs of common viewpoints: up-front and up-rear, up-right-front and up-left-rear, up-left-front and up-right-rear, right and left. As a result, the training samples are merged from the original 11 categories to five, and models are learned on five viewpoints accordingly, i.e., *k* = 5. These are labelled as up, left/right, up-front/up-rear, up-right-front/up-left-rear, and up-left-front/up-right-rear. 

### 4.1. Deformable Part Model

The vehicle model for the *k*th viewpoint is based on a star model of a pictorial structure and is described by a root model and several parts models. A set of permitted locations for each part with respect to the root is combined with the cost of deformation to each part. Formally, it is an n + 2 tuple, as defined by Equation (3):

(3) $$M_{D}=\{F_{0},(F_{1},v_{1},d_{1}),\ldots ,(F_{n},v_{n},d_{n}),b\}\;$$

where $F_{0}$ is the root filter, *n* is the number of parts $\left(P_{1},P_{2},\ldots P_{n}\right)$ and *b* is the bias term. Each part model *P_i_* is defined by a 3-tuple (*F_i_*, *v_i_*, *d_i_*), where *F_i_* is the *i*th part filter, *v_i_* is a bidimensional vector that specifies the fixed position for part *i* relative to the root position, and *d_i_* is a four-dimensional vector that specifies the coefficients of a quadratic function defining a deformation cost for each placement of part *i* relative to *v_i_*. The score is given by the following formula:

(4) $$score(p_{0},\ldots p_{n})=\sum_{i=0}^{n}{F^{\prime }}_{i}\cdot \phi (H,p_{i})-\sum_{i=0}^{n}d_{i}\cdot \phi_{d}(dx_{i},dy_{i})+b\;$$

where $\phi (H,p_{i})$ is a subwindow in the space-scale pyramid H with the upper left corner in *P_i_*. $\left(dx_{i},dy_{i})=(x_{i},y_{i})-(2(x_{0},y_{0})+v_{i}\right)$ gives the displacement of the *i*th part relative to its anchor position and $\phi_{d}(dx_{i},dy_{i})=(dx_{i},dy_{i},dx_{i}^{2},dy_{i}^{2})$ are deformation features.

### 4.2. CRF Model 

Occlusion can be defined in DPM by a grammar model; however, it is time consuming. Niknejad [5] provided a method using DPM and CRF as a two-layer classifier to detect occluded vehicles. In the first layer, DPM generates root and parts scores and the relative configuration for parts. In the second layer, the CRF uses the output from the DPM to detect the occluded objects. 

#### 4.2.1. Occlusion State

**Figure 5:**
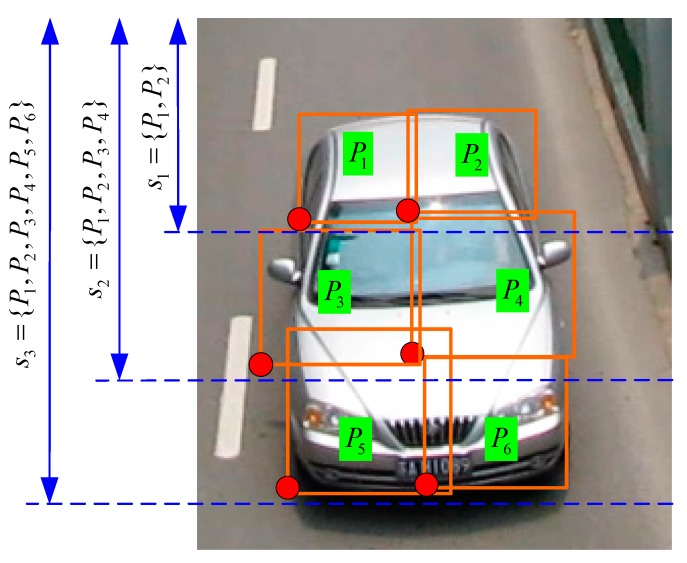
Parts clustering based on the occlusion direction.

The occlusion states are finite variables relating to the viewpoints and can be defined based on the visibility of root and part filters $\left(P_{1},P_{2},\ldots P_{n}\right)$. We sum up all possible occlusion states for each viewpoint. Figure 5 depicts an example of parts clustering of a vehicle up-front model from up to down direction. The occlusion state *s_j_* has the character of $s_{j-1}\subset s_{j}$.

#### 4.2.2. CRF Model

**Figure 6:**
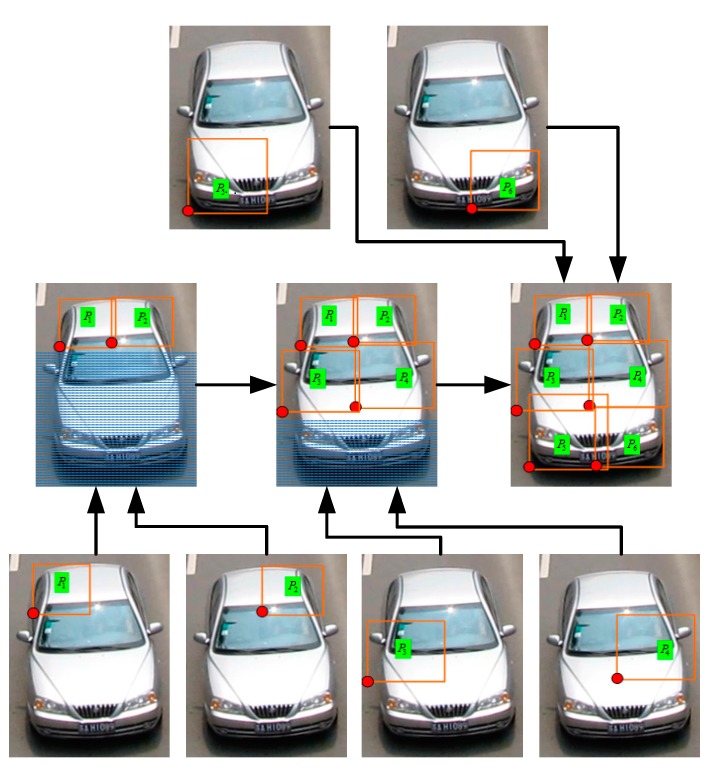
CRF model for up-front vehicle model.

{*P_i_*} are set to be the nodes in lowest layer of the CRF, and {*s_j_*} are set to be other nodes in the CRF, as shown in Figure 6. Given a bounding box *i* with label *y_i_*, we can calculate the detection conditional probability by maximizing the probability over all occlusion states *s_j_*:

(5) $$P(y_{i}|v_{i};\theta )=\underset{j}{\arg\max}(P(y_{i},s_{j}|v_{i};\theta ))\;$$

where $v_{i}$ is the features vector and θ is a CRF parameter. $v_{i}$ is generated by the DPM and includes the scores for parts and best possible placement of the parts. The probability $p(r^{k}|M_{C}^{k})$ with the *k*th viewpoint is converted into $P(y_{i}|v_{i};\theta )$ with labeled vehicle *y_i_* in the *k*th viewpoint.

In the CRF, the maximum of $P(y_{i},s_{j}|v_{i};\theta )$ can be converted to get the largest energy function $\psi (y,s_{j},v;\theta )$ over all occlusion stages. ψ(⋅) contains energy from both parts and their relations.

(6) $$\psi (y,s_{j},v;\theta )=\sum_{\forall p_{k}\in \Omega }f(y,p_{k},s_{j},v)\cdot \theta^{n}\;+\sum_{\forall p_{k},p_{l}\in \Omega }g(y,p_{k},p_{l},s_{j},v)\cdot \theta^{c}\;$$

where $\Omega =\{p_{0},\ldots p_{n}\}$, $\theta^{n},\theta^{c}$ are the components of $\theta =(\theta^{n},\theta^{c})$ in the CRF model and correspond to the part information and the relative spatial relation with other parts, respectively, and f(⋅) features depend on a single hidden variable in the CRF model, while g(⋅) features depend on the correlation between pairs of parts.

A belief propagation algorithm is generated to estimate each occlusion state. The detection conditional probability is as follows:

(7) $$Z(y|v,\theta )=\arg\max_{j}(\exp(\psi (y,s_{j},v;\theta )))\;$$

Then the detection probability can be calculated by:

(8) $$P(y|v,\theta )=Z(y|v,\theta )/\sum_{\hat{y}}Z(\hat{y}|v,\theta )\;$$

Using a Bayesian formula, the likelihood of each occlusion state is calculated as follows:

(9) $$P(s_{j}|y,v,\theta )=P(y,s_{j}|v,\theta )/P(y|v,\theta )\;$$

where P(y|v,θ) is constant for all occlusion stages and calculated by maximizing over all possible occlusion states [5]. The marginal distribution for visibility over each individual part $p_{k}=a$ or pairs of parts corresponding to edges in the graph is calculated as follows:

(10) $$P(s_{j},p_{k}=a|y,v,\theta )\approx f(y,p_{k}=a,s_{j},v)\cdot \theta^{n}\;$$

(11) $$P(s_{j},p_{k}=k,p_{l}=l|y,v,\theta )\approx g(y,p_{k}=k,p_{l}=l,s_{j},v)\cdot \theta^{c}\;$$

For the root $p_{0}$, the energy is calculated by concatenating the weight vectors of the trained root filter and HOG(Histogram of Oriented Gradient) feature at the given position:

(12) $$f(y,p_{0}=0,s_{j},v)=F_{0}\cdot \phi (H,p_{i})\;$$

For parts $\left(P_{1},P_{2},\ldots P_{n}\right)$, the score of parts is summed according to their appearance in the direction view as the following functions:

(13) $$f(y,p_{k}=k,s_{j},v)=\sum_{h=0}^{k}\underset{dx,dy}{\max}(F_{h}\cdot \phi (H,(x_{h}+dx,y_{h}+dy)p_{i})-d_{k}\cdot \phi_{d}(dx,dy))\;$$

For the spatial correlation, a normal distribution is used through the following formula:

(14) $$\begin{array}{l} g(y,p_{k}=k,p_{l}=l,s_{j},v)\cdot \theta^{c}=\theta_{(k,j)x}^{c}\cdot N(\Delta_{l,k}^{x}|\mu_{l,k}^{x},\sigma_{l,k}^{x})\;\\ \;+\theta_{(k,j)y}^{c}\cdot N(\Delta_{l,k}^{y}|\mu_{l,k}^{y},\sigma_{l,k}^{y}) \end{array}$$

where $\Delta_{l,k}^{x}$, $\Delta_{l,k}^{y}$ are the relative positions of parts $p_{k}$, $p_{l}$ in the *x* and *y* directions.

(15) $$\Delta_{l,k}^{x}=(x_{p}-x_{l}),\;\Delta_{l,k}^{y}=(y_{p}-y_{l})\;$$

$\theta_{(k,j)x}^{c}$,$\theta_{(k,j)y}^{c}$ are parameters that correspond to the relative spatial relation between parts $p_{k}$, $p_{l}$ in the *x* and *y* directions, respectively. $\mu_{l,k}^{x},\sigma_{l,k}^{x}$ are mean and covariance values that correspond to the relative spatial relation between parts $p_{k}$, $p_{l}$ in the *x* direction that was extracted for all positive samples from the DPM training data.

#### 4.2.3. Model Learning

For each viewpoint k, the root filter $F_{0}^{k}$ and part filters $F_{i}^{k}$, i=1,…n are learned using the image samples of the category. Here, n is set as a preassigned constant value, which simplifies the calculation. The MLE (maximum likelihood estimation) method is used to estimate the parameters $\theta^{*}=\arg\max_{\theta }L(\theta )$ from the training samples.

## 5. Viewpoint Detection by Spatial Context-Based Inference

Normally, distributions on where, when, and what types of vehicle activities occur have a strong correlation with the road structure, which is defined by road geometry, number of traffic lanes, and traffic rules. Given a type of road structure, the viewpoint of a subject vehicle at any specific location can be predicted by the scene model. 

### 5.1. Scene Modeling

A scene model can be manually described or automatically extracted from the static scene appearance. On the other hand, a scene model can be extracted from regular vehicle motion patterns, e.g., trajectories, which tends to be a better method than the other two methods. 

#### 5.1.1. Trajectory Coarse Clustering

During an uncongested traffic period, general vehicle trajectories can be obtained from coarse vehicle trajectories via a blob tracker based on background subtraction and length-width ratio constraints. Through a period of observation, it is possible to obtain thousands of trajectories from a scene. 

Before clustering, some outliers caused by tracking errors must to be removed. Trajectories with large average distances to neighbors and with very short lengths are rejected as outliers. After that, the main flow direction (MFD) vector x is used to group the trajectories:

(16) x=((x(t)−x(0)),(y(t)−y(0))) 

where (x(0),y(0)) and (x(t),y(t)) are the position vectors of the starting point and ending point of a trajectory, respectively.

Each cluster is described as a Gaussian function with a mean $\mu_{k}$ and covariance matrix $\sigma_{k}$. The overall distribution p(x) considering all MFD vectors can be modeled as a mixture of Gaussians (MoG):

(17) $$p(x)=\sum_{k=1}^{k_{\max}}\omega_{k}p_{k}(x)\;$$

Here, $k_{\max}$ is the number of classes, $\omega_{k}$ is the prior probability, and $p_{k}(x)$ is the normal distribution for the *k*th class. Then, each vector x in the training period is assigned to a class according to

(18) $$k=\underset{j\in \{1,\ldots k_{\max}\}}{\arg\max}\omega_{j}p_{j}(x)\;$$

The number of Gaussians in the mixture is determined by the number of clusters.

#### 5.1.2. Classification Filtering

The detailed filtering procedure was developed in the authors’ previous work [27]. First, clusters that are significantly broader than the remaining clusters are removed:

(19) $$|\sigma_{m}|>2\sum_{i}\left|\sigma_{i}\right|\;$$

where $\sigma_{m}$ is the determinant of the covariance matrix of the *m*th cluster and $\sigma_{i}$ is that of any other.

Second, clusters that have obvious overlaps are merged. Here, the Bhattacharyya distance–based error estimation method is used [28]. The expected classification error E (in %) between two classes is defined as

(20) $$E=40.22-70.02b+63.58b^{2}-32.77b^{3}+8.72b^{4}-0.92b^{5}\;$$

where *b* describes the Bhattacharyya distance. In this paper, the threshold is set to 1.5. Then, if the Bhattacharyya distance b<1.5, the two clusters will be merged.

Lastly, isolated outliers are removed. Such error is generated by incorrect tracking due to occlusion or formed when overlaps split. In such cases, the maximum and minimum peak coefficients based adaptive mean and variance estimation method can be used [29].

#### 5.1.3. Trajectory Fine Clustering

After coarse clustering, trajectories moving in opposite directions are separated, regardless of their relative spatial proximity. The road structure of the scene can be represented by path models with entries, exits, and paths between them. However, trajectories with similar moving directions are clustered regardless of the magnitude of their relative spatial separation. During the fine clustering procedure, each class of trajectories is further clustered according to different spatial distributions.

The trajectory similarity is defined by the Hausdorff distance, which considers the location relationship between trajectories and is a simple algorithmic calculation.

Considering two trajectories $A=\{{\vec{a}}_{i}\}$ and $B=\{{\vec{b}}_{i}\}$, where ${\vec{a}}_{i}=<x_{i}^{a},y_{i}^{a}>$, ${\vec{b}}_{i}=<x_{i}^{b},y_{i}^{b}>$ are the spatial coordinates, for an observation ${\vec{a}}_{i}$ on A, its nearest observation on B is

(21) $$d_{E}(a_{i},b_{j})=\underset{j\in B}{\arg\min}||(x_{i}^{a}-x_{j}^{b},y_{i}^{a}-y_{j}^{b})||\;$$

**Figure 7:**
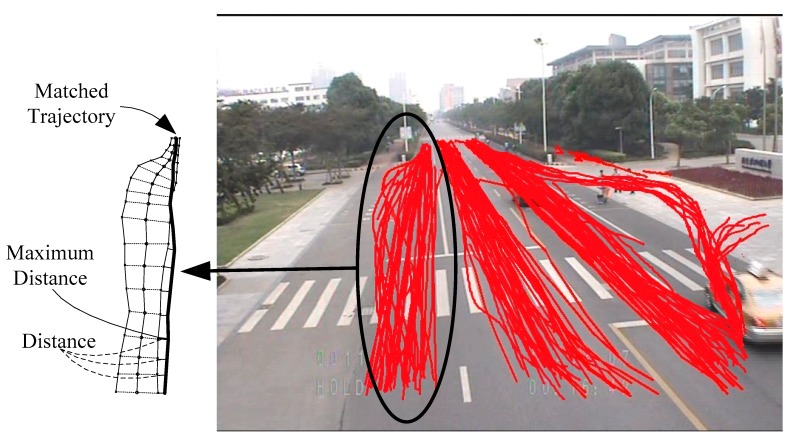
Matched trajectory. The maximum distance of the trajectory from the route model is smaller than the allowed threshold.

The Hausdorff distance between A and B is

(22) $$D_{H}(A,B)=\max(D_{h}(A,B),D_{h}(B,A))\;$$

where $D_{h}(A,B)=\underset{i\in A}{\max}(d_{E}(a_{i},b_{j}))$. A summary of the fine clustering procedure is as follows:

(1) The first trajectory of the dataset initializes the first route model.
(2) Other trajectories are compared with the existing route models. If the calculated distance by Equation (12) is smaller than threshold τ, the route model is updated (as shown in Figure 7). Otherwise, a new model is initialized.
(3) If two route models are sufficiently overlapped, they are merged.

### 5.2. Viewpoint Inference

#### 5.2.1. Some Phenomena in Imaging

**Figure 8:**
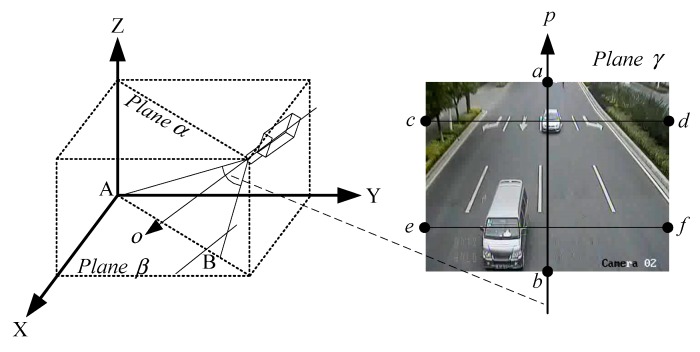
Camera model and corresponding transform from 3D world plane to 2D image plane.

As show in Figure 8, according to the imaging principle, plane α, determined by the camera's optical axis ο, and projection point P of the camera's center on ground plane β correspond to the center line p in the image plane γ. Let $\bar{AB}$ be the line segment of the interaction of the camera’s field of vision (FOV), plane α, and β. Then, $\bar{ab}$ is the corresponding line segment in plane γ.

(1) When a car goes from point A to point B, its image in plane γ starts from point *a* to point *b* with viewpoints from up-front to up.
(2) When a car appears in the camera’s far FOV and comes along line segment $\bar{cd}$, the viewpoint is right.
(3) When a car is in the camera’s near FOV and comes along line segment $\bar{ef}$, the viewpoint is up.

In conclusion, vehicle viewpoint is directly determined by location and motion direction, which can be described by the trajectory gradient.

#### 5.2.2. Inference of Viewpoint Probability Distribution

**Figure 9:**
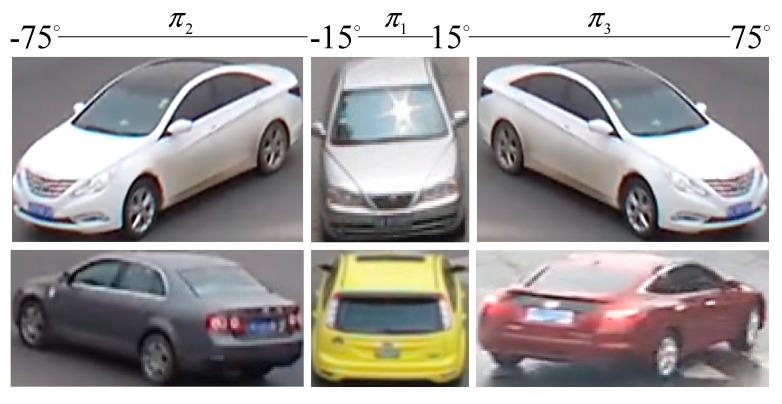
Division of viewpoints under middle field of view (FOV).

In the far FOV, the vehicle area is small and its viewpoints can be treated as two kinds, left/right and up-front/up-back. In the near FOV, the vehicle viewpoint is set to be up. In the middle FOV, the viewpoints of vehicles are divided by trajectory gradient, as shown in Figure 9, where region $\pi_{1}$ is in the up-front/up-rear viewpoint, region $\pi_{2}$ is in the up-left-front/up-right-rear viewpoint, and region $\pi_{3}$ is in the up-right-front/up-left-rear viewpoint.

**Figure 10:**
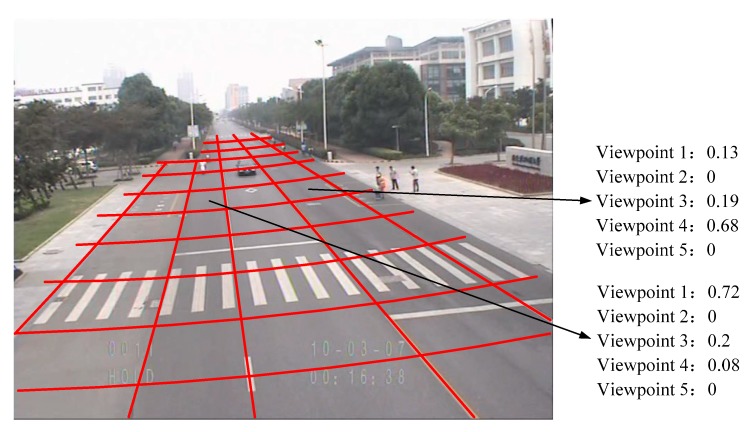
Result of viewpoint probability distributions.

For a fixed location $r=(x_{r},y_{r})$, if it is located in N path models, then the probability of vehicle viewpoint *i*, i=1,2,…5, in *r* is

(23) $$p(r^{i}|T_{s})=\sum_{t=1}^{N}q_{t}(i)/\sum_{t=1}^{N}M_{t}\;$$

where $M_{t}$ is the number of total trajectories passing through the cross-section with *r* in model *t* and $q_{t}(i)$ is the number of trajectories with viewpoint *i* in the *t*th model. An example of the detection result of viewpoint probability with a stationary camera is shown in Figure 10.

#### 5.2.3. Implementation

The classification of near FOV, middle FOV, and far FOV can be done according to the camera parameters and the size of the vehicle; however, it can be difficult to obtain the relevant parameters for a fixed camera needed to help determine the appropriate FOV region. In this paper, a simple division method is used that classifies the top one-sixth of an image as without consideration for the target area, the next one-fifth from the top is defined as the far FOV of the camera, the bottom one-fifth is set to be near FOV for the camera, and the remainder of the middle of the image is defined as the middle FOV for the camera.


**Pseudocode of algorithm**
1 Function **fusingpartbasedObjectRecognition ()**2 Build image pyramid      **for** s = 1 to *k*        I_S_*G→*I_G_*        calumniate *I_S+1_* by subsampling *I_S_* with factor λ       **next**3 Build HOG feature pyramid      **for** s = 1 to *k*         calumniate gradient orientation image and gradient magnitude image         **for** y = 1 to *H* step 8           **for** x = 1 to *W* step 8            set H′(x,y,s) as gradient orientation histogram based on *I_Ψ__,S_* and weighted by *I_Mag__,S_*              **next**         **next**         calculate H(x,y,s) through normalization of H′(x,y,s) with respect to four blocks containing current cell      **next**4 Calculate matching score functions      **for** s = 1 to *k*       **for** y = 1 to *H* step 8         **for** x = 1 to *W* step 8            **for** i = 1 to *M*               calculate m_i_(x,y,s,u_i_)               **if**
*i*≠1 **then**               generalized distance transform calculate *P_i_(x,y,s)* for subsequent backward DP step               end if             **next**           **next**         **next**       **next**5 Find all local max Find all local maximal of root part above similarity threshold

## 6. Experimental Results

### 6.1. Dataset

A dataset consisting of thousands of image samples used for offline vehicle model training was generated from video sequences obtained from the surveillance system in our laboratory and the Internet. Some images from a Caltech dataset [30] were used as an implementation. Vehicles were first detected in the video sequences based on background subtraction and length-width ratio constraints. Second, vehicles were checked and labeled by hand. The positive samples were from the above three sources, whereas the negative samples were from random background images. The positive samples were divided into five categories according to the vehicle’s viewpoints, which again are up, left/right, up-front/up-rear, up-right-front/up-left-rear, and up-left-front/up-right-rear.

We used more than 10 scenarios for the actual testing environment. As shown in Table 2, the testing samples cover a broad range of traffic conditions, from sparse to dense traffic, containing vehicles with different viewpoints and shapes, and under different illumination conditions. In particular, several experiments on congested traffic conditions are represented.

### 6.2. Experiment Details

The hardware part of the system was built of the image acquisition device, server, and client. The image acquisition device uses a network camera, and the highest resolution is 1920 × 1080, high definition, and no delay. The system uses its SDK (software development kit) development kit to achieve video image sequence acquisition. The server is used for video processing and the client is a PC. The software part of the system consists of two parts: the server and the client. The server includes an acquisition server and a central server. The acquisition server sends the video data captured by the camera to the central server. The central server performs real-time vehicle detection or query results according to the received client commands. At the same time, the test result or query result is sent to the client and displayed on the client interface.

The number of training samples was 28,600, of which 80% (22,880) were used for training and 20% (5720) were used for verification, and the total number of testing samples was 40,126. The testing samples were independent of the training samples. 

### 6.3. Experimental Results 

The presented results include a contrasting quantitative experiment on the labeled testing images and several experiments on complex urban traffic conditions.

#### 6.3.1. Quantitative Experiment

In the quantitative experiment, we divided the testing samples into two sets of fully visible and partially occluded vehicles, which accounted for 22,206 and 17,920 samples, respectively. 

**Figure 11:**
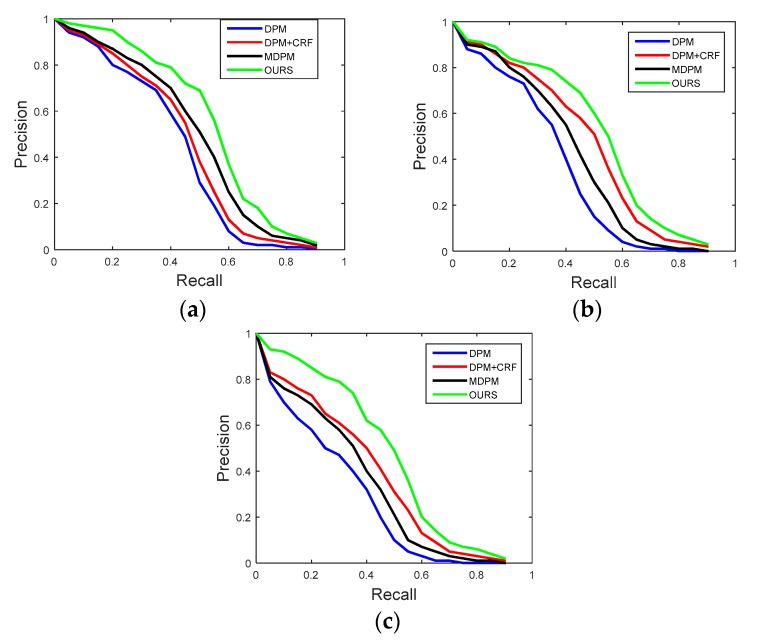
Performance comparison of precision–recall curves for four methods. (**a**) Comparison of fully visible vehicles; (**b**) comparison of partially occluded vehicles under low traffic density; (**c**) comparison of partially occluded vehicles under high traffic density.

A vehicle was determined to be detected only when the overlap between its bounding box and the ground truth was greater than 50%. The recall and precision of our method on fully visible vehicles, as well as the results from three other methods, are shown in Figure 11a. The results of recall and precision for partially occluded vehicles under low traffic density and high traffic density conditions are shown in Figure 11b,c, respectively. Four approaches are compared, which makes up the proposed work we describe, the deformable part model (DPM) [1], the deformable part model with conditional random field model (DPM + CRF) [5], and the multiple deformable part model (MDPM) [3].

For a particular threshold, precision and recall are (true positive)/(true positive + false positive) and (true positive)/(true positive + false negative), respectively. It can be observed from Figure 11 that the approach we propose demonstrates comparable results to the other methods. The increased number of viewpoints and viewpoint map helped to improve the detection rate by filtering out other confusing background objects and estimating vehicle poses. The average processing time for the four methods (DPM, DPM + CRF, MDPM, OURS) was 23.7 ms, 32.3 ms, 29.1 ms, and 33.9 ms, respectively, for an input image of 640 × 480 scale with C/C++ code optimization and acceleration.

For stationary cameras in video surveillance, the combination of viewpoint inference improves the performance of pattern recognition–based vehicle detection methods. In the following figures, we show detailed detection results for different viewing conditions.

**Figure 12:**
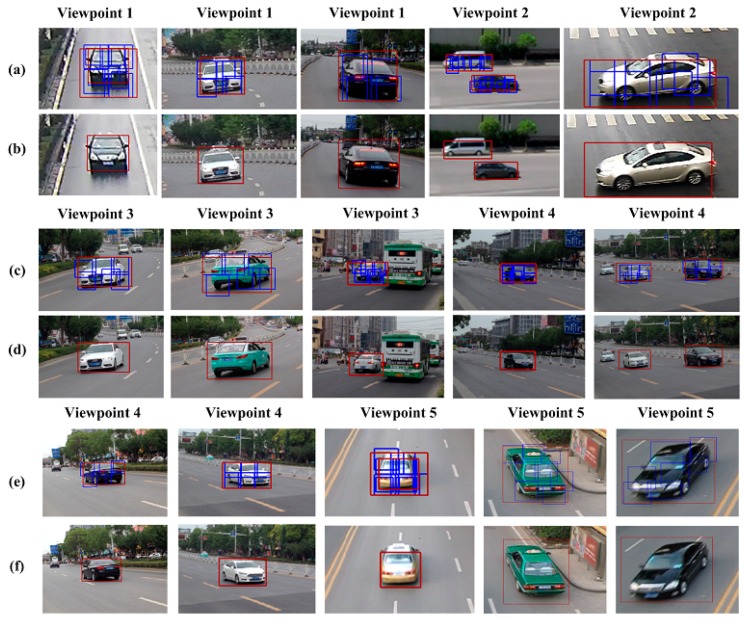
Vehicle detection results for five viewpoints. (**a**) vehicle parts detection results in viewpoint1-viewpoint2; (**b**) vehicle global detection results in viewpoint1-viewpoint2; (**c**) vehicle parts detection results in viewpoint3-viewpoint4; (**d**) vehicle global detection results in viewpoint3-viewpoint4; (**e**) vehicle parts detection results in viewpoint4-viewpoint5; (**f**) vehicle global detection results in viewpoint4-viewpoint5.

Figure 12 provides vehicle detection results for five viewpoints, where the blue boxes denote detected vehicle parts and the red box denotes the final vehicle detection result. The results demonstrate that our method can accommodate different vehicle shapes and poses. Further, the method demonstrates good detection of nonoccluded vehicles in low-density traffic conditions. It is noted that since the training images did not include buses, our method failed to detect buses in the test data.

**Figure 13:**
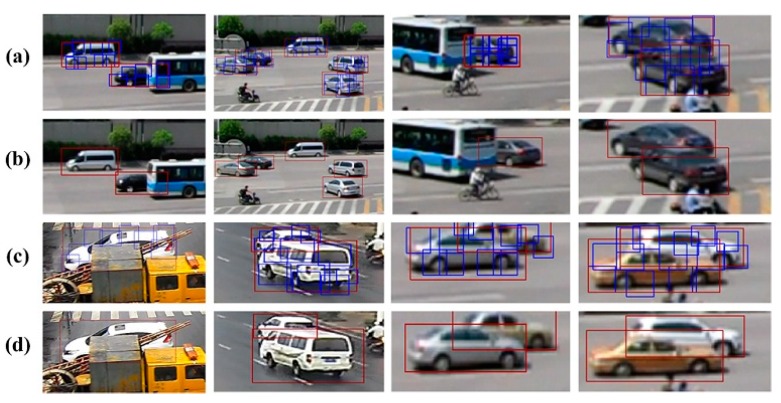
Vehicle detection results with occlusion. (**a**) vehicle parts detection results in partial occlusion scenario 1; (**b**) vehicle global detection results in partial occlusion scenario 1; (**c**) vehicle parts detection results in partial occlusion scenario 2; (**d**) vehicle global detection results in partial occlusion scenario 2.

As a result of the loss of depth information in the image projection transformation from 3D to 2D, occlusion happens frequently in video surveillance with monocular stationary cameras. Thus, our testing images include partial occlusions. As shown in Figure 13, the proposed method can detect partially occluded vehicles with similar shapes, as long as more than 20% of the vehicle body is observable.

**Figure 14:**
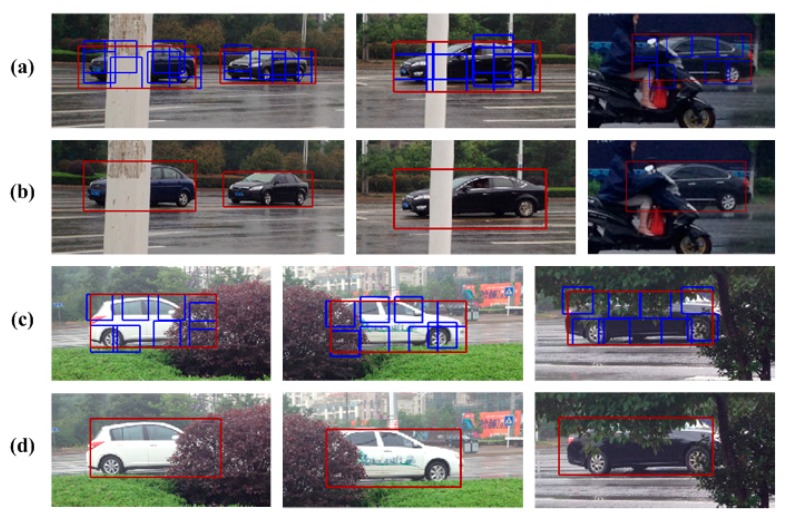
Vehicle detection results with occlusion on a rainy day. (**a**) vehicle parts detection results in partial occlusion scenario 1 of rainy day; (**b**) vehicle global detection results in partial occlusion scenario 1 of rainy day; (**c**) vehicle parts detection results in partial occlusion scenario 2 of rainy day; (**d**) vehicle global detection results in partial occlusion scenario 2 of rainy day.

In actual traffic scenes, the vehicle may also be obscured by road infrastructure as well as other common road features. As shown in Figure 14, the occluded vehicle parts can be deduced by the CRF model, which improves the model score of DPM and guarantees the detection rate of occluded vehicles. 

In addition to the vehicle poses and shapes, the test images also include different weather conditions, consisting of sunny, cloudy, rainy, and twilight conditions. Our method is not affected by shadows on a sunny day, and can detect vehicles in rainy and twilight situations, both of which result in poor image quality. However, our method fails to detect vehicles at night or in heavy fog conditions, because the vehicle edges are nearly invisible in these conditions. Our method will be further improved to address these limitations in our future work. 

#### 6.3.2. Experiments in Congested Traffic Conditions

**Figure 15:**
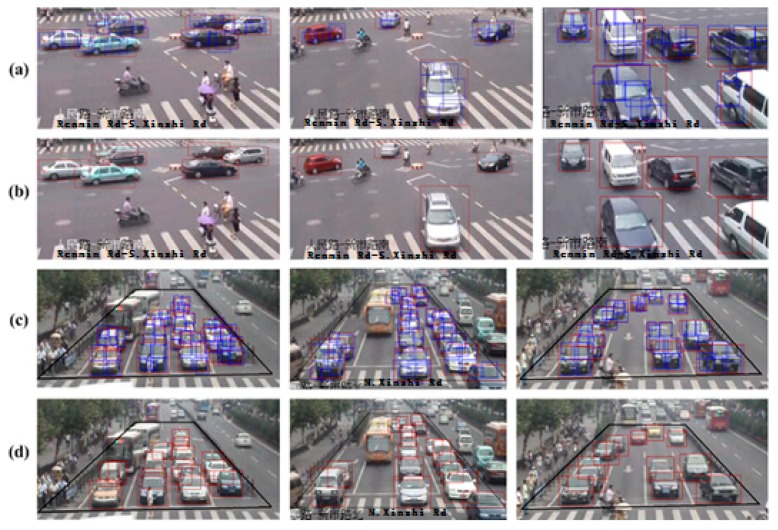
Vehicle detection results in congested traffic scenes. (**a**) vehicle parts detection results in congested traffic scene 1; (**b**) vehicle global detection results in congested traffic scene 1; (**c**) vehicle parts detection results in congested traffic scene 2; (**d**) vehicle global detection results in congested traffic scene 2.

As described previously, congested traffic conditions bring challenges to the effectiveness of vehicle detection methods. Therefore, we purposely paid attention to studying congested traffic conditions. In high-density traffic flow, the continuous adhesion of vehicles greatly affects the detection rate. As shown in Figure 15a, there is no consistent viewpoint distribution for a fixed camera location at intersections, so the detection result is given by the DPM + CRF. As shown in Figure 15c, five vehicles occlude each other in the middle FOV image region. With our method, the occlusion states contain only two vehicle parts in the CRF model in the up-front view. As a result, the five vehicles are all detected. Our proposed algorithm performs well at a certain resolution in the region of interest. However, in the far-field region, the area of the vehicle is very small and the edge detection effect is not ideal, leading to a low detection rate for the low overlapping rate between the estimated bounding box and the real vehicle region.

#### 6.3.3. Experimental Analysis

From experiments on various proposed road environments for the proposed method, from the test results, it can be seen that in a rainy environment with multitarget detection, the algorithm has a good detection effect, and also has better detection performance for vehicles with different perspectives in the actual acquisition video. However, due to the relationship of the training set, it is impossible to smoothly detect vehicles such as buses, trucks, etc., and this relationship is prone to false detection. The practical application shows that the proposed method can solve the problem in vehicle detection. Under normal lighting conditions, the detection rate is high, is not affected by most of the shadows, and is not sensitive to the background. It can complete most of the vehicles on the road with occlusion detection, and has better detection performance.

In future work, we will attempt to make our method more suitable for real-world traffic video surveillance systems. First, further computation acceleration may be realized by leveraging parallel processing. Second, an adaptive parameter estimation method can be developed to avoid manually setting relevant parameters. Finally, it is necessary to expand our method to more object types by deep part hierarchies or by building more object models.

## 7. Conclusions

In this paper, we propose a vehicle detection method by combining scene modeling and deformable part models for fixed cameras, particularly for use in congested traffic conditions. The proposed method consists of constructing a DPM + CRF model for each viewpoint to represent vehicles, training scene models, viewpoint inference, and vehicle object detection by addressing both partial observation and varying viewpoints within a single probabilistic framework.

Compared with the current methods, there are two main innovations in this paper. First, we use the scene context information to constrain the possible location and viewpoint of a vehicle, which is reasonable since the camera is often in a fixed location in a real traffic scene. In practical applications, the judicious usage of scene information can potentially eliminate some interference and reduce required computation times. Second, based on the combined DPM and CRF model, the viewpoint-related vehicle models are built and the occlusion states are defined individually. Compared to the MDPM, the proposed method provides semisupervised viewpoint direction, which performs well for a variety of traffic surveillance conditions. Experimental results demonstrate the efficiency of the proposed work on vehicle detection by a stationary camera, especially for solving the occlusion problem in congested conditions.

## Figures and Tables

**Table 1:** Pros and cons of related work.

Methods	Pros	Cons
Simple features-based methods [6,7,8,9]	Easy to describe and perform in specific applications	Can only be used in specific simple scenes; cannot handle occlusion
Manual part-based model-based methods [10,11,12,13,14,15,16,17,18]	Able to handle weakly partial occlusion	Still low-detection performance in complex scenes
Deformable part-based model-based methods [1,5,19,20,21,22,23,24,25,26]	Improved performance in vehicle detection	Cannot handle heavy occlusion

**Table 2:** Testing sample details.

		Fully Observed	Partially Observed
**Viewpoint**	1	5800	4580
	2	980	3600
	3	7420	5700
	4	6750	4022
	5	1256	18
**Traffic density**	Low	18,016	5380
	High	4190	12,540
**Total**		22,206	17,920
